# “Always paracetamol, they give them paracetamol for everything”: a qualitative study examining Eastern European migrants’ experiences of the UK health service

**DOI:** 10.1186/s12913-017-2526-3

**Published:** 2017-08-29

**Authors:** Hannah Madden, Jane Harris, Christian Blickem, Rebecca Harrison, Hannah Timpson

**Affiliations:** Public Health Institute, Liverpool John Moores University, Liverpool, UK

**Keywords:** General practice, European Union, Migrants, Eastern Europe, Primary care, United Kingdom, Antibiotics, Paracetamol/Acetaminophen, NHS/National Health Service

## Abstract

**Background:**

The enlargement of the European Union since 2004 has led to an increase in the number of Eastern European migrants living in the UK. The health of this group is under-researched though some mixed evidence shows they are at higher risk of certain physical health conditions such as heart attacks, strokes, HIV and alcohol use and have poorer mental health. This is compounded by poor or insecure housing, low pay, isolation and prejudice. We aimed to understand the health needs and health service experiences of the Eastern European population in a town in Northern England.

**Methods:**

Five semi structured one-to-one and small group interviews and five focus groups were conducted with 42 Eastern European participants between June and September 2014. The majority of participants were Polish and other participants were from Belarus, Hungary, Latvia, Russia, Slovakia and Ukraine. The data were analysed using thematic framework analysis.

**Results:**

Key findings included a good understanding the UK health service structure and high registration and use of general practice/primary care services. However, overall, there were high levels of dissatisfaction, frustration and distrust in General Practitioners (GP). The majority of participants viewed the GP as unhelpful and dismissive; a barrier to secondary/acute care; reluctant to prescribe antibiotics; and that GPs too often advised them to take paracetamol (acetaminophen) and rest.

**Conclusions:**

Overwhelmingly participants had strong opinions about access to primary care and the role of the general practitioners. Although the design of the UK health service was well understood, participants were unhappy with the system of GP as gatekeeper and felt it inferior to the consumer-focused health systems in their country of origin. More work is needed to promote the importance of self-care, reduce antibiotic and medication use, and to increase trust in the GP.

## Background

A significant increase in the numbers of foreign-born people in the UK was seen between 2005 and 2008 following the European Union (EU) Enlargement in 2004 [1]. In 2014, the year when most recent figures are available, Poland represented the largest proportion of foreign-born citizens living in the UK (15.1% of all foreign-born citizens); Romania (3.5%) and Lithuania (3.3%) were also in the top ten countries of citizenship for foreign citizens in the UK [1].

Migrants coming to work in the UK are often thought to be healthier as they tend to be younger, on average, than the general UK population [2]; an idea which is often referred to as the ‘healthy immigrant effect’ [3]. However, evidence about the health status of Eastern European migrants to the UK and disease burden in this group comes from small-scale local needs assessments or using small samples of less rigorously collected local data [4–8]. Although some evidence suggests that people living in Eastern Europe may be at increased risk of heart attacks, strokes, HIV/STIs, and alcohol abuse [9–13], evidence on physical and mental ill-health and alcohol problems experienced by Eastern European migrants to the UK are mixed [4, 8, 14, 15]. Studies have suggested that international migrants to the UK are less likely to be admitted to hospital, although the criteria used to presume residency status means these findings must be interpreted with caution [16].

Historically much of the research on the experience and use of health services among migrants and ethnic minorities in the UK has focused on the longer established black and minority ethnic (BME) communities of Caribbean, Indian, Pakistani, Bangladeshi and Chinese migrants [17]. As a relatively new group of migrants to the UK, there is limited research on how Eastern Europeans experience the NHS. It is likely that similar language barriers, ‘cultural differences’ and unfamiliarity with the NHS system could exist for all migrants to the UK. Therefore, research on other migrant groups in the UK may shed light on the experience of Eastern European migrants. Previous research has shown a slightly higher use of general practice (GPs) by Caribbean, Indian, Pakistani and Bangladeshi patients and lower use of hospital services by Indian, Pakistani, Bangladeshi and Chinese groups [18]. Despite this increased use of primary care, studies show Asian patients have greater difficulty in accessing their GP than non-Asian patients [19] and south Asian and Chinese patients report a less positive experience of primary care [20]. Language and cultural misunderstandings are common in GP consultations, across patients with many countries of origin [21] and may contribute to this dissatisfaction. Access to hospital care and consultants has also been reported as an issue for BME and migrant groups. For example more Asian than non-Asian patients would prefer direct access to consultants [19] and asylum seekers in Scotland report frustration at lack of direct access to specialist care and are not accustomed to a system where the GP acts as gatekeeper [22]. Furthermore, Atkinson et al. [23] found ethnic minorities had little understanding of the role of pharmacists or other community healthcare providers.

Barriers to accessing health services for Eastern European migrants may include lack of knowledge regarding how the UK health system is organised, and the role of the GP and primary care [6, 8, 24]. This lack of knowledge affects use, expectation and experience of primary care [25]. Whilst migrants with fluent English [24] or who those who have been in the UK longer [26] have more understanding of the NHS, a lack of information can still lead to inappropriate use of services. For example, it has been reported some Eastern European patients tend to attend accident and emergency (A&E) departments instead of the GP because they may not understand the UK health system or may find it hard to register with a GP [5]. Evidence about GP registration levels is mixed [6, 25–27] but individuals who were not registered cited ignorance of the process and language as the main obstacles [6]. Data collected by Osipovič in 2007 and 2008 show Polish patients in London focus on self-care and delay seeking primary and secondary care, often because they are unsure what NHS treatment they could access for free [26].

A lack of understanding of the system can lead to dissatisfaction, frustration and distrust in the NHS, and the GP in particular. In the London study, Polish patients felt the GP was not authoritative enough and the focus on person-centred care in the UK led them to believe GPs were not knowledgeable or well-trained [26]. In another example, Greenhalgh described her professional experience and how her Polish patients believe doctors in Poland take illness more seriously and make more specialist referrals, that postnatal care continues for longer periods, and sick children can access a paediatrician quicker [28]. Their dissatisfaction and distrust of the GP led Greenhalgh to feel like a “*travel agent*” who Polish patients are trying to “*get past in order to access proper medicine*” [28]. Travelling back to Poland for medical treatment (both primary and secondary) also appears to be common [26, 27, 29].

Many Eastern European migrants are not accustomed to a model of healthcare where the GP is the gatekeeper to other services and may anticipate direct access to hospital and secondary care services, and a lower threshold for certain screening procedures and investigations than in the UK – for example, expecting more scans in pregnancy [26, 30]. Expectations of prescribing and the availability of medicines are also different and may be due to variations in the culture of healthcare and clinical practice in participants’ countries of origin [7, 25]. For example, some research with recent Polish migrants revealed participants’ frustration with GPs recommending paracetamol as treatment [28] with participants referring to the GP as the “*paracetamol force*” [26] and the “*paracetamol service*” [27].

There is currently only limited research that explores Eastern European migrants’ experiences of healthcare services in the UK, and particularly primary care. The main large study on this topic by Osipovič collected data in London in 2007 and 2008 and focused only on Polish migrants [26]. With a growing Eastern European population across the whole of the UK, it is important to understand their attitudes to health and their experiences of health care services. There is a particular lack of evidence on how experiences may differ between London and smaller towns, or between north and south England. This study is set in a medium sized industrial town in the north of England. As primary care is an important access point to a number of health and social care services, this study explores Eastern European migrants’ experiences of healthcare services, including primary care, in the UK to understand perceived barriers to engagement.

It is important to note that over the next 3 years, there are likely to be changes to the rights to NHS care and patterns of health services use by Eastern European migrants. This will come about as a result of the June 2016 referendum vote for the UK to leave the EU. These changes are currently very unclear and this research was conducted before the referendum so reflects the experience of the participants prior to possible political changes.

## Methods

### Sampling and recruitment

Focus groups, one-to-one interviews and small group interviews were used to gather a comprehensive understanding of the health experiences and needs of the Eastern European population in an medium town in Northern England. In total 42 individuals participated in the research (Table 1). Three quarters of participants were Polish (*n* = 28), which is broadly representative of the Eastern European population in the area [31]. Fourteen participants came from other countries including Belarus, Hungary, Latvia, Russia and Ukraine.

**Table 1:** Participant demographics

Data collection method (no. participants)	Gender	Age Range	Nationalities	Label
Youth Focus Group (*n* = 10)	Eight males and two females	Aged 16–19	All Polish	Youth focus group
Mothers 1 Focus Group (*n* = 6)	Six females	Aged 18–65	All Polish	Mothers 1
Mothers 2 Focus Group (*n* = 4)	Four females	Aged 18–65	All Polish	Mothers 2
Mothers 3 Focus Group (*n* = 10)	10 females	Aged 25–45	All Russian speaking. Mix of Russian, Latvian, Ukrainian and Belarussian	Mothers 3
Mixed focus group (*n* = 5)	Three males and two females	Aged 25–60	All Polish	Mixed focus group
One-to one- interview	Female	60s	Polish	F1
One-to one- interview	Female	50s	Polish	F2
One-to one- interview	Male	Early 20s	Polish	M1
Small group interview (n=3)	Three males	Aged 21–35	Latvian	M2, M3, M4
One-to one- interview	Male	30s	Hungarian	M5
Total = 42				

Purposive sampling was used to recruit participants to interviews and focus groups. Advertisement materials were provided in English and Polish. Adverts were displayed in local Polish and Eastern European networks including: Polish and Romanian grocery shops, churches, online forums and a Polish Saturday school. Recruitment was also promoted through general services such as social housing providers, local authority services, recruitment agencies, a drug and alcohol service and libraries. A variety of advertisement methods were used with the organisations listed above including posters, leaflets and mailshot via email lists held by organisations. Snowballing techniques were used to increase recruitment to interviews.

Advertisement materials and participant information sheets were provided in English and Polish. Potential participants were invited to email or telephone the researchers for information and offered information sheets in other languages if they wanted; no other languages were requested. Participants who responded to the adverts were offered the option of a one-to-one interview or a paired interview, or to take part in a focus group with their friends.

To encourage open and easy conversation, in only one language, we only conducted focus groups with participants who already knew each other. Four of the focus groups were recruited through key community members or local government employees acting as gatekeepers, distributing recruitment materials and inviting potential participants. Participants who were part of existing support or community groups (the mothers group at a children’s centre, the Polish Saturday School and the Polish Youth Group) were invited to take part during their normal meeting time. We approached all groups that were known to the local government and key community members and all agreed to take part. The fifth focus group was a participant-recruited group of family and friends.

The final sample was dependent on who responded to the recruitment materials and volunteered to take part in the study. We invited participants to self-identify as Eastern European (for example one gatekeeper felt there was a debate over whether Poland was in Central or Eastern Europe). Unfortunately, despite the very wide-ranging recruitment strategy, no participants from Romania or Lithuania agreed to part and the researchers were unable to find any formal groups to invite for a focus group. It is not possible to establish the response rate as no participants declined to take part; however, we expect many people will have seen the advertisements but not contacted the researchers.

All potential participants who contacted the researcher and those who were part of formal groups were provided with a detailed participant information sheet at least a week before the focus group/ interview. This included information on the purpose of the study, researcher credentials, procedure and any risks and benefits to the participants. Written consent was obtained from all participants. For each of the existing groups being invited to a focus group, a gatekeeper was provided with a gatekeeper information sheet and provided written consent for us to approach their group members.

### Data collection

The first and second authors are experienced and trained female qualitative researchers and conducted all the data collection. There was no existing relationships between participants and researchers. The focus groups were conducted in English and participants assisted each other with any translation issues. Each focus group lasted between 30 min and 1 h and were conducted in the groups normal meeting place, one focus group was conducted in the participant’s home. The participant’s young children were present in the room during two of the mothers’ focus groups.

Face-to-face interviews were conducted by the first or second author in a quiet area in a suitable local venue chosen by the participants (including cafes and community centres). Interviews were conducted in English and lasted between 30 min and 1 h. All only took part in one type of data collection and participants were given a £10 shopping voucher to thank them for their time. Data were collected between June and September 2014.

### Focus group and interview discussion topics

A focus group and interview discussion guide was developed to explore health need. This was informed by the literature review and engagement with local stakeholders. Topics for discussion included: perceptions of health; attitudes and health behaviours in the Eastern European community including mental health, alcohol and smoking; awareness of health services including GPs, hospitals, dentists and pharmacies; any experience and opinions of local health services; barriers to accessing health services; how to reduce barriers to access for Eastern European people to public health services; and how to target health promotion services.

### Data analysis

Focus groups and interviews were audio recorded and transcribed verbatim from the recordings. All identifiable information was removed and anonymity and confidentiality maintained. Framework analysis was used to analyse the data [32], and was conducted by the lead author and checked by the second author. Framework analysis begins inductively with a pre-set group of aims and is considered particularly appropriate for policy related or applied qualitative research [33].

A mixture of pre-set and open codes were applied to the first four transcripts and a matrix (or framework) developed from these. Subsequent transcripts were then indexed onto the matrix using these existing categories and codes [34]. Although some themes were identified in advance, based on the discussion guide, some (for example paracetamol) developed from the data. The interpretation of the framework is presented with illustrative quotes to highlight key findings. Unfortunately, it was not possible to return transcripts and findings to participants for verification due to time limitations.

### Language and translation

All recruitment materials (posters and leaflets), the participant information sheets and consent forms were translated into Polish by a native speaker. Gatekeepers and participants were asked if they would like these translated into other languages when interviews and focus groups were arranged, however, this was not requested.

All interviews were conducted in English with any issues about unknown words being resolved between the interviewer and participant using rephrasing and hand gestures. The focus groups were conducted mainly in English with occasional translation by other members of the groups.

### Ethics and quality

All participants and gatekeepers were supplied with information sheets and written consent was provided by all. Ethical approval was granted by Liverpool John Moores University Research Ethics Committee (ref: 14/EHC/042). A COREQ checklist was completed and submitted with this manuscript [35].

## Results

The role of the GP was only one element of the discussion guide; however, it became clear in the early stages of data collection that this topic was extremely important to participants with the conversation often returning to experiences of primary care and the role of the GP. Consequently, this topic was explored with all participants to understand how their experience of the GP impacted on their overall health and wellbeing and their experience of the UK health system as a whole. It was very difficult to encourage participants to talk about their attitudes and perceptions of health and public health services (for example smoking and alcohol) and the discussion always returned to their experiences of the GP. Only a small minority of participants spoke about these other topics and the findings were inconsistent, therefore it would be irrelevant and inappropriate to include these in this publication. The themes that emerged came from the priorities of the participants and were: navigating the UK health system; experience of GPs and primary care, paracetamol (acetaminophen) and rest; and becoming accustomed to UK health services.

### Navigating the UK health system

Although the vast majority of participants were registered with a GP, most felt it was difficult to understand the health system when they first arrived in the UK and all participants reported it was very different to the health system in their home country. Some participants told how they were instructed by employers to register with a GP but were not offered support with the process and that this information is hard to find:

*“'You need to register with a GP’. That is not telling you anything. How? What is a GP?” (M5)*

*“Yes, everybody understands [the role of GP] and also if you go to work, taking a job they asking me, ‘are you registered with GP?’ Because if there’s any accident or anything, they want people to be registered with the doctors.” (F1)*

Friends, family and work colleagues who were already established in the UK played an important role in helping new migrants understand how services worked and how to register with a GP.

*“Just by chatting to other people and the longer you stay then you understand better.” (female, Mothers 3)*

There was low awareness of and use of pharmacy services with most participants saying they visited their GP if they or their children are unwell and the GP was the main source of information on health. Awareness of other services such as smoking cessation, sexual health, mental health and drug and alcohol services were very low.

### Experience of general practitioners and primary care

The majority of participants reported having a good understanding of the design of the UK health service, including the role of the GP in referring to specialist services and secondary/acute care, however, they disliked the system. There was much discussion about the differences between health systems in the UK and participants’ country of origin, and the perceived inferiority of the UK system. Much of this dissatisfaction was focussed on the role of the GP who participants felt did not take their health complaints seriously and appeared dismissive, uninterested and short on time. Many participants felt they were an inconvenience to the GP, that they were wasting the GP’s time, and the general advice to go home and allow their illness to get better in time was the GP wanting them to leave their office. Examples provided by participants where this had happened included illnesses such as adult chicken pox, childhood asthma, childhood dermatology, high fevers and back pain. A minority of participants believed this attitude was because the GP was trying to save money for the NHS.

*“Sometimes I feel like they [GP] are saying ‘why are you coming to see me?” (M5)*

*“It is just ‘next’ [dismissive hand gesture] ‘next’” (female, Mixed focus group)*

This perceived lack of interest was particularly frustrating for parents who felt the GP did not take concerns about their children seriously; children with a high temperature or with a severe cough were told they would get better on their own. This did not reassure parents and some reported that they resorted to visiting the hospital accident and emergency department. Many participants discussed the difficulty of booking a GP appointment at short notice although it was acknowledged that this varied between practices.

*“It is not possible, last time my son he have five days his temperature 41 [Celsius, 106 Fahrenheit] it was really high and they said ‘sorry but we don’t have any place for him and call Tuesday, Wednesday and Thursday’. After that I’m going to the hospital cos they don’t have any place for me” (Mothers 1)*

Many participants discussed how the GP could block access to a specialist in the UK whilst in Eastern Europe they could access many more health services directly. Some participants also appeared to resent the power held by the GP and wanted to be able to self-refer to secondary (hospital) care when they felt it necessary. Some participants discussed how they had specifically asked to be referred to services such as gynaecology or endocrinology and their GP had refused. There was a lot of frustration that there is no alternative way to access specialist services other than by GP referral or privately. Many of the mothers felt they had to return to the GP regularly with the same problem to ‘prove’ how ill their child was before the GP would refer them to a specialist.

*Participant 3: “To get the specialist you need to prove it long period.”*

*Participant 5: “But really if you’ve got some symptoms which are really worrying…”*

*Participant 3: “You have to prove it long but with asthma or something they will go straight away maybe to hospital…” (Mothers 1)*

*“We said problem first contact the doctor because the first time they [GP] ignoring you, they say ‘nothing wrong with you, nothing wrong with you’, but after when they find what’s going on [care improves]…” (Mothers 2)*

Some participants felt that the consultations with GPs in the UK were too rushed, did not physically examine patients and only relied on description of symptoms. This was particularly difficult if patients did not have the English skills to describe their symptoms. Some participants suggested that the GP needed to spend more time examining and understanding their condition before dismissing them – it was felt that telephone consultations (without seeing a patient) were inappropriate.

*“Ah, here doctors are so rushed, if you have a cold they don’t even bother, don’t even make an appointment with you... Just wait for a couple of weeks, cough may last for six weeks, in Russia, they always prescribe medicine.” (Mothers 3)*

*“It’s er the conversation between the doctor and the personal is bigger [in Poland]…listening and talking and…take care and took me a lot of time …But er in England, in my opinion, cos this, this and this [quick hand motions] and go” (Mothers 2)*

Some participants believed the doctors in their home countries were better trained, had more knowledge and were more competent and would seek out Polish dentists and GPs in the UK. A small minority of participants reported their friends would pay privately for a consultation with a Polish doctor. However, some acknowledged that the facilities and equipment in the UK is of a higher standard than their home country.

*“[In Poland] knowledge is better, medicine study is very difficult” (Female, mixed focus group)*

### Paracetamol and rest

Feeling dismissed by their GP was the strongly linked to GP prescribing habits. Participants’ general feeling was that GPs usually advised patients to take paracetamol (also known as acetaminophen or Tylenol) and would not prescribe any other medication.

All focus groups and all but one interview participant mentioned their dissatisfaction with the common suggestion of “*paracetamol and rest*” that they receive from their GP. Participants reported that this was something they would discuss with their friends from Eastern Europe. When one focus group participant mentioned paracetamol, often, the other participants would laugh and repeat the phrase “*always paracetamol*”, as if it was a common joke. They felt that they always received this same advice and that GPs did not take their concerns seriously. They felt that GPs should prescribe more antibiotics and some described a struggle to convince the GP to prescribe them stronger drugs.

*“They give you paracetamol, they always do. Headache paracetamol. Stomach pain paracetamol. Temperature paracetamol” (Male, youth focus group)*

*“It’s just when you go whatever you ask they just say ‘have paracetamol’….very typical….Paracetamol that’s it…Treats every single thing” (Mothers 3)*

Participants were frustrated as they felt they had wasted time attending the GP practice just to be told to take medication they have at home. The expectation that the GP will only prescribe paracetamol discouraged some participants from visiting their GP. In particular, participants wanted to understand why this advice was being given; it was not just that they were unused to UK prescribing practices but also that they received no explanation to help them understand why paracetamol was the most appropriate treatment.

*“The smallest [child] had a temperature of forty degrees [104 Fahrenheit] he [GP] said to give paracetamol. That is not an explanation, ‘he have fever give paracetamol’. But why?” (M5)*

* Participant: “If you have sniffs or something go to doctors, if you have really very high temperature or infection, but they say ‘ahh take paracetamol for three weeks’”*

*Researcher: “Do you think that this idea that you’re kind of just given paracetamol and you’re sent home stops people going [to the GP]?”*

*Participant: “Yeh, you still go but maybe what would be helpful, not just ‘oh, there’s nothing wrong with you’ to explain why the person may be worried, just giving more explanation.” (Mothers 3)*

Many participants suggested that it was easier to access medication (especially antibiotics) in their country of origin, where they had been prescribed specific drugs for their health complaints and they did not understand why these were unavailable in the UK.

*“Always paracetamol...they give them paracetamol but they’re taking paracetamol for everything, for everything. [In Latvia] they give you medicine for headache, you’ve got stomach pain they give you medicine for stomach pain but [in UK] they just give paracetamol.” (M2)*

### Settling in: Becoming accustomed to UK health services

A minority of participants felt that the UK’s approach to medication and promotion of self-care was appropriate. They did suggest this was difficult to understand when first arriving in the UK, however, once people had lived in the UK a number of years they understood that the UK system prioritises self-care, prevention and minimal use of drugs where possible. Three women (one Polish, one Russian and one Ukrainian) in two focus groups felt Eastern European health services are too reliant on antibiotics and dominated by the pharmaceutical industry. A fourth female interview participant, who was Polish, felt that the Polish community want a “*miracle*” and quick, easy fix to problems, which led to an over-reliance on medication. However, these views were very much the minority and the women expressing these views all had high English language proficiency, were educated to university level and had lived in the UK over 10 years.

*“It is getting crazy now, cos it’s free and now everything is going medicine they want to sell it in Poland and want to recommend everyone, the doctor want to give you everything all the time, medicine, medicine, medicine and with pregnancy is really crazy now, really crazy…” (Mothers 2)*

*“This is different cos in England your body have to help itself you know, it’s a different way of thinking, you wait and your body should help yourself...They don’t help you too much. If you feel really bad they will help you but in Poland they help all the time with medicine, medicine, medicine….” (Mothers 1)*

## Discussion

Prescribing practices, GP behaviour and attitudes were the main concerns of participants in this study who began discussing GPs as soon as they were asked any questions about their understanding of health and being healthy. Although the discussion guide was broad and included questions on attitudes and perception of health and on public health services, throughout the focus groups and interviews, the conversation regularly returned to primary care and prescribing, even when researchers were asking seemingly unrelated questions. Discussions of overall perceptions and attitudes to health were difficult to encourage and so brief and that they were rarely evident in the analysis.

Almost every participant reported they felt their GP regularly did not take their concerns seriously and advised them to “*take paracetamol and rest*”. This caused a lot of frustration, and some amusement, within the Eastern European community and many participants spoke about how their friends and colleagues reported the same experience. Although this issue is mentioned in the existing literature, the ubiquity of this feeling and the importance to the Eastern European community in this research cannot be understated. Many participants reported feeling frustrated that their GP had not prescribed antibiotics when requested and some thought the GP was trying to save money or did not believe their illness was serious. This frustration was particularly evident amongst mothers accessing healthcare for their children and these findings concur with a recent study by Sime [29], which suggests that mothers arriving in the UK from Eastern Europe are particularly active in evaluating their decisions about healthcare access and are not merely passive receivers of services. Greenhalgh also reported that Polish patients in the UK believe UK GPs “just give out paracetamol” [28].

The concern with over-reliance on paracetamol and perception of reluctance by the GP is not unique to the Eastern European community in the UK. Another UK study about the experiences of GP antibiotic prescribing to recent migrants found participants from Sierra Leone, Sudan, Zimbabwe, Iran and Poland were all frustrated by the GP advising paracetamol [36]. A study of Somali refugees’ experiences of the Dutch healthcare system [37] also found the same frustration. In both these studies, and in a study of a variety of migrants accessing primary care in Birmingham [38], participants interpreted the suggestion of paracetamol and rest as their GP not taking them seriously. The literature highlights higher antibiotic resistance in Eastern Europe compared to the UK [39] and a lack of understanding of the appropriateness of antibiotics in both the UK national and asylum seeking populations [22, 40]. Specific promotion may need to target the Eastern European community in the UK to stop an increase in antibiotic resistance.

This difference in ethos and approach to healthcare is causing distrust between the Eastern European population and their GP thus creating barriers to primary care, which could also deter patients from accessing other healthcare services. Sime et al. [29] found a similar experience when talking to health professionals and recent Eastern European migrants in Scotland; differences in provision had led to disappointment for migrants when they could not self-refer to a specialist or had a long waiting time for an appointment. Similar findings have been found with asylum seekers in the UK [22]. This new model of care with the GP as gatekeeper could lead to inappropriate service use with some participants reporting bypassing their GP and attending A&E. If patients feel dismissed and not listened to by their GP they are unlikely to approach their GP for support for issues such as mental health, alcohol or smoking cessation which is particularly pertinent as knowledge of other community based health services was low. There was also evidence in this study of overreliance on the GP with very few participants using pharmacy services. This has important wider public health implications as the GP may represent not only the main source of clinical care, but also the sole source of public health advice and intervention for the majority of participants.

The participants in this study had high rates of GP registration though they felt that it had been challenging to understand how to register when they first arrived in the UK. Peers were felt to be key sources of advice and guidance on the NHS and some participants felt employers should do more to support GP registration. Employers of large numbers of Eastern European workers may be positioned to provide advice and signposting and encourage migrant workers to register with a GP during induction.

This distrust in the GP and the ethos of the UK health system is not unique to the Eastern European community but are an important example of a wider issue amongst many migrant groups. Although some of these negative experiences reported may be due to GP behaviour, participants’ perception of disinterest and dismissal by their GP suggests the problem is twofold. Firstly, the Eastern European communities appear to access GP services in a role of consumer, expecting treatment for issues and illnesses that can be treated at home or by a pharmacist. Experience in their country of origin may have led them to expect a consumer-focused health service where the GP provides medication on request for all health concerns and participants reported more varied and strong medication being prescribed with no resistance. Secondly, the GP has a role to play in building trust and promoting both the importance of self-care and the efficacy of paracetamol. Generic paracetamol cost as little as £0.50 (€0.70) for sixteen tablets of 500mg and, without relevant information from their GP, the affordability and availability of paracetamol seems to create the perception among the Eastern European community that paracetamol is not an effective drug. Promotion of self-care needs be targeted at this population, possibly through national campaigns.

Previous research has found that lack of understanding by Eastern European migrants to the UK leads to inappropriate use of services instead of their GP [6, 8, 24]; however, this was not the case for the participants in this study. The majority of participants were registered with a GP and understood the role of the GP in the UK health service. However, as mentioned in the literature [28, 29], the GP was often seen as a barrier; they were seen to hold the power to refer or deny specialist services and many participants talked about having to ‘prove’ how ill they were before they could be referred. Greenhalgh reported that new Polish migrants “*brought memories of a healthcare system in which general practice is often the last refuge of the failed physician, and the middle classes access specialist care without encountering significant gatekeeping hurdles*.” [28]. The literature states this dissatisfaction and frustration is a result of not understanding the UK health system and prescribing practices [7, 25], however, it is important to stress this study found that Eastern Europeans generally report they have a good understanding of the UK health service. Therefore, it is not that they do not understand how the system is organised, it is that they do not like the system and think it is inferior to the system to which they are accustomed.

There are some clear implications for UK policy makers and practitioners, and some issues that need to be overcome if Eastern European migrants are to engage more appropriately with primary care and better understand notions of self-care. The reasons for the design of the UK health system and the ethos behind the NHS need to be better communicated and explained to the Eastern European community, in appropriate languages, explaining how to access primary, secondary and acute health services. Also required is improved provision of self-care information and education. Vitally, primary care practitioners need to build trust with the Eastern European community, not just provide education and information.

### Limitations

Participants from numerous countries of origin with both new and established UK residency provides a breadth of understanding. However, differences in experience and understanding may exist between different nationality groups and newly arrived versus established communities which are not reflected in this study. Experiences with GPs were universally mentioned in this cohort and data saturation felt to be reached. However, Eastern European populations in different parts of the UK may have different experiences of primary care given the variation in both attitudes to migration and approaches to health service delivery across the UK. Participants mainly self-selected for this study replying to adverts and invites and may have been influenced by the £10 voucher. This was a qualitative research study in a medium sized town in Northern England with a mainly white British population; we must acknowledge these findings may not be generalisable to other areas. Our sample was mainly Polish and although findings were similar across participants, these may have differed if the distribution of nationalities had been different. We were unable to recruit participants from all Eastern European countries. Although efforts were made to minimise the impact of translation, the budget for the study did not include interpretation services. The majority of the participants had quite good spoken English, however, some of the nuance may have been lost due to language barriers. Additionally those migrants with the highest needs may be the least proficient in English and this study may underrepresent this group.

## Conclusions

This study highlights a good understanding of the design of UK health services by Eastern European migrants in a medium sized town in Northern England. However, the participants were dissatisfied with primary care services; they felt their illnesses were not taken seriously; they were not prescribed the medication they felt they needed; and were too often advised to take paracetamol and rest. The majority of participants believed the UK health system was inferior to their home system. This study builds on previous research and highlights that it is not simply a lack of understanding of health service design that is leading to dissatisfaction. The key issue is the difference between UK and home systems and a dislike of the UK ethos, which promotes self-care and limited use of antibiotics. Further information about why, not just how, the UK health service is delivered and the importance of person - centred care and self-care is needed to increase satisfaction and promote appropriate use of services in this population.

## Abbreviations

A&E: Accident and Emergency (UK)
COREQ: Consolidated Criteria for Reporting Qualitative Research
EU: European Union
GP: General Practitioner/General Practice
HIV: Human Immunodeficiency Virus
NHS: National Health Service
STI: Sexually Transmitted Infection
UK: United Kingdom
